# Nontuberculosis mycobacteria are the major causes of tuberculosis like lesions in cattle slaughtered at Bahir Dar Abattoir, northwestern Ethiopia

**DOI:** 10.1186/s12917-017-1168-3

**Published:** 2017-08-15

**Authors:** Anwar Nuru, Aboma Zewude, Temesgen Mohammed, Biniam Wondale, Laikemariam Teshome, Muluwork Getahun, Gezahegne Mamo, Girmay Medhin, Rembert Pieper, Gobena Ameni

**Affiliations:** 1 Aklilu Lemma Institute of Pathobiology, Addis Ababa University, P.O. Box 1176, Addis Ababa, Ethiopia; 2 Animal Diseases Investigation and Diagnostic Laboratory, Amhara Region Bureau of Agriculture, P. 0. Box 70, Bahir Dar, Ethiopia; 3 College of Veterinary Medicine and Agriculture, Addis Ababa University, P.O. Box 34, Debre Zeit, Ethiopia; 4 Ethiopian Public Health Institute, P. O. Box181689, Addis Ababa, Ethiopia; 5 College of Veterinary Medicine and Animal Sciences, University of Gondar, P.O. Box 346, Gondar, Ethiopia; 6 J. Craig Venter Institute, 9704 Medical Center Drive, Rockville, MD USA

**Keywords:** Cattle, Nontuberculosis mycobacteria, TB-like lesion

## Abstract

**Background:**

The main cause of bovine tuberculosis (bTB) is believed to be *Mycobacterium bovis* (*M. bovis*). Nontuberculosis mycobacteria (NTM) are neglected but opportunistic pathogens and obstacles for bTB diagnosis. This study aimed to isolate and characterize the mycobacteria organisms involved in causing TB-like lesions in cattle in northwestern Ethiopia.

**Results:**

A total of 2846 carcasses of cattle were inspected for TB lesions. Ninety six tissues (including lymph nodes such as submandibular, retropharyngeal, tonsilar, mediatinal, bronchial and mesenteric, and organs such as lung, liver and kidney) with suspicious TB lesion(s) were collected and cultured on Lowenstein-Jensen medium. Twenty one showed culture growth, of which only 17 were identified containing acid fast bacilli (AFB) by Ziehl–Neelsen staining. Among the 17 AFB isolates 15 generated a polymerase chain reaction product of 1030 bp by gel electrophoresis based on the 16S ribosomal RNA gene amplification. No *M. tuberculosis* complex species were isolated. Further characterization by Genotype Mycobacterium CM assay showed 6 isolates identified as *M. peregrinum*. Eight isolates represented by mixed species, which includes *M. fortuitum-peregrinum* (3 isolates), *M. gordonae-peregrinum* (3 isolates) and *M. fortuitum-gordonae-peregrinum* (2 isolates). One NTM could not be interpreted.

**Conclusion:**

A significant number of NTM species were isolated from TB-like lesions of grazing cattle slaughtered at Bahir Dar Abattoir. Such finding could suggest the role of NTM in causing lesions in cattle. Further investigations are recommended on the pathogenesis of the reported NTM species in cattle, and if they have public health significance.

## Background

Bovine tuberculosis (bTB), which is primarily caused by *Mycobacterium bovis* (*M. bovis*) is an endemic disease of cattle in Ethiopia and distributed in almost all parts of the country. Although its current prevalence rate, at a national level, is unknown, previous Ethiopian studies have shown that the average herd prevalence of bTB in smallholder farms is 21.1% [1–4] and intensive dairy production systems is 49.3% [5, 6]. Other Ethiopian studies [7–15] undertaken at abattoirs have reported bTB in cattle based on TB-like lesion with an estimated average prevalence of 5.57%. In addition, *M. bovis* was also recovered from TB lesions in cattle, spoligotyped, and their strain types were identified and reported by previous studies in Ethiopia [7, 9, 16–19]. Infection with *M. bovis* can be transmitted from cattle to humans, mainly through the consumption of contaminated milk and meat [20], although there is no evidence that this has happened in Ethiopia, where raw milk and meat consumption is widely habituated. Although the *M. tuberculosis* complex (MTBC) species are identified as strict pathogens of TB in human and animals, other mycobacteria species collectively referred to as nontuberculosis mycobacteria (NTM) also play a significant role as a source of infections [21]. However, there have been no studies to date conducted to identify the specific species of NTMs that are causing TB lesions in cattle in northwest Ethiopia. Presently fast, easy and sensitive molecular tools are available for the detection and identification of MTBC and NTM [22]. Thus, identification of mycobacteria is required using these molecular tools to guide therapy and for epidemiological purposes.

In the present study NTMs were predominantly isolated and characterized from TB-like lesions of cattle by molecular tools such as mycobacterium genus typing and Genotype Mycobacterium CM assay. *M. peregrinum* was the most dominant NTM species recovered from 6 isolates. Eight isolates represented by mixed species such as *M. fortuitum-peregrinum* (3 isolates), *M. gordonae-peregrinum* (3 isolates) and *M. fortuitum-gordonae-peregrinum* (2 isolates). One NTM could not be interpreted even if it had a band pattern of 1,2,3 and 10, and no MTBC species were identified.

## Methods

### Description of the study area and setting

The study was conducted in cattle slaughtered at Bahir Dar Abattoir, which is located in Bahir Dar City of Amhara Regional State, northwest Ethiopia. Currently, Bahir Dar Abattoir is the only licensed slaughter house in Bahir Dar City, which fulfils the daily beef requirements of over 200,000 inhabitants of the city, peri-urban areas and its neighboring rural villages. Cattle slaughtered at the abattoir were mainly of the Zebu type and originated from different districts of Amhara Region and the neighboring Oromia Region (Amhara and Oromia regions are among the nine ethnically based regional states of Ethiopia, and have the largest number of livestock and human population compared to other regions).

### Sample collection and processing

A total of 2846 cattle slaughtered from October 2014 to December 2015 at Bahir Dar Abattoir were thoroughly inspected for TB lesions. Parotid, mandibular, retropharyngeal, tonsilar, left and right bronchial, cranial and caudal mediastinal, brochial, tracheobronchial and mesenteric lymph nodes, and organs including the lungs, liver and kidneys were examined. The seven lobes of the two lungs were inspected externally and palpated. Each lobe was sectioned into approximately 2 cm thick slices to identify the lesions. Similarly, lymph nodes sliced into sections of a similar thickness and inspected for the presence of visible lesions. The animal was classified as having lesion when gross lesion(s) suggestive of bTB were found in any of the tissues examined. Each specimen was processed and cultured for the isolation of mycobacteria following standard procedure described by OIE [23]. In brief, the tissue samples were manually dissected in to small pieces and homogenized using a pestle and mortar. The homogenate decontaminated by an equal volume of 4% NaOH and concentrated by centrifugation at 3000×g for 15 min. The sediment was neutralized with 2 N HCl using phenol red as an indicator, and inoculated onto Lowenstein Jensen (LJ) glycerol and LJ pyruvate solid media slants. The culture media were incubated at 37 °C for 8 weeks, and considered negative if no visible growth was detected after the eighth week of incubation. Ziehl–Neelsen (ZN) staining microscopic examination was performed to select acid fast bacilli (AFB) positive isolates. Presumptive mycobacterial colonies were heat-killed at 85 °C for 45 min by mixing ∼2 loop-full of cells in 200 μl distilled H_2_O for further molecular activities.

### Mycobacterium genus typing

Multiplex polymerase chain reaction (mPCR) using six oligonucleotide primers was performed as described previously [24]. Primer pairs included were MYCGEN-F 5′-AGA GTT TGA TCC TGG CTC AG-3′, MYCGEN-R 5′-TGC ACA CAG GCC ACA AGG GA-3′, which amplify a specific PCR product from the 16S rRNA gene of all know mycobacteria were used. MYCAV-R 5′-ACC AGA AGA CAT GCG TCT TG-3′ and MYCINT-F 5′-CCT TTA GGC GCA TGT CTT TA-3′ which amplify the hyper variable region of the 16S rRNA gene of *M. intracellulare* (MYCINT-F) and *M. avium* (MYCAV-R), respectively. Two primers (TB1-F 5′-GAA CAA TCC GGA GTT GAC AA-3′) and (TB1-R 5′-AGC ACG CTG TCA ATC ATG TA-3′), which target for the MPB70 gene were used to specify *M. tuberculosis* complex from the mycobacteria.

Amplification was done as recommended. In each run *M. avium* and *M. bovis* were included as a positive control with sterile water (H_2_O Qiagen) as a negative control. The PCR products were electrophoresed in 1.5% agarose gel, and the final image visualized under ultraviolet light.

### The GenoType® mycobacterium common Mycobacteria (CM) assay

The GenoType® Mycobacterium CM assay (Hain Lifescience, Nehren Germany) was used to analyze NTM isolates at the species level, and the procedure described in the manual enclosed in the kit was followed to conduct the test. The assay involved DNA amplification targeting the 23S rRNA gene region, as recommended. Followed by the reverse hybridization to specific oligonucleotide probes immobilized on membrane strips, which was conducted on a shaking TwinCubator (Hain). The final result was interpreted based on the presence and absence of bands, and compared with the evaluation sheet provided with the kit. *M. tuberculosis* H37Rv, *M. fortuitum* and *M. abscessus* were used as appositive control while H_2_O Qiagen as a negative control.

### Ethical considerations

The study was approved by Ethical Review Board (Ref. number IRB/05-02/2013) of the Aklilu Lemma Institute of Pathobiology, Addis Ababa University. Study permission also obtained Amhara Region Bureau of Agriculture Department of Animal Agency, and Municipality Office of Bahir Dar City.

## Results

### Description of the study animals and tissues

The vast majority of studied cattle was male (88.7%, 2524/2846) and zebu breed (99.9%, 2842/2846). Seventy nine carcasses had lesion(s) suspected of bTB resulting in an overall animal level prevalence of 2.78% (79/2846). The animal level prevalence was defined as the number of cattle positive for TB-like lesion(s) per 100 cattle examined. From 79 positive cattle a total of 96 different tissues having TB-like lesions were collected, processed and cultured onto LJ media. Of which 21 showed culture growth, and only 17 colonies were identified containing mycobacteria by ZN staining with an overall AFB positivity of 17.7% (17/96). The 17 mycobacterial isolates were detected only from 12 slaughtered cattle, and the largest proportion was observed in the retropharyngeal lymph nodes (75%) followed by submandibular and the kidney tissues (each with 50% proportion). The type and number of tissues identified with suspicious TB lesion(s), and their corresponding AFB positivity are indicated in Table 1.

**Table 1:** Cattle tissues identified with suspicious tuberculosis lesions and mycobacteria

Type of tissue	Number of tissue with lesion(s)	^a^AFB positive, +n(%)
Submandibular lymph node	2	1(50.0)
Retropharyngeal lymph node	4	3(75.0)
Tonsilar lymph node	3	1(33.3)
Mediastinal lymph node	12	1(8.33)
Bronchial lymph node	10	1(10.0)
Mesenteric lymph node	43	3(7.00)
Lung tissue	6	2(33.3)
Liver tissue	14	4(28.6)
Kidney tissue	2	1(50.0)
Total	96	17(17.7)

^a^Acid fast bacilli; +number of tissues positive for AFB; The 96 tissues suspected of having tuberculosis lesion(s) were identified from 79 of 2846 cattle slaughtered at Bahir Dar Abattoir, northwestern Ethiopia. Of which 17 tissues identified containing mycobacteria by Ziehl-Neelsen staining and were recorded from carcasses of only 12 cattle

### Identification and speciation of nontuberculosis mycobacteria

**Fig. 1:**
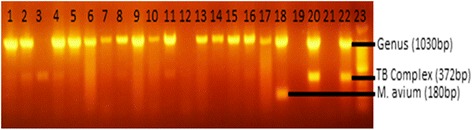
Gel electrophoresis of PCR products from AFB isolated from cattle tissue containing TB-like lesion(s). The Seventeen acid fast bacilli positive tuberculosis lesions were identified from 79 tissues of 2846 cattle slaughtered at Bahir Dar Abattoir, northwest Ethiopia. Lanes 1-17 = test isolates, Lane 18 = *M. avium* (positive control), Lane 19 = missed out, Lane 20 = *M. bovis* (positive control), Lane 21 = Qiagen H_2_O (negative control), Lane 22 = *M. tuberculosis* (positive control), and Lane 23 = 100 bp DNA ladder

Among the 17 AFB positive mycobacterial isolates 15 generated a PCR product of 1030 bp by gel electrophoresis (Fig. 1), and consequently identified as NTM.

**Fig. 2:**
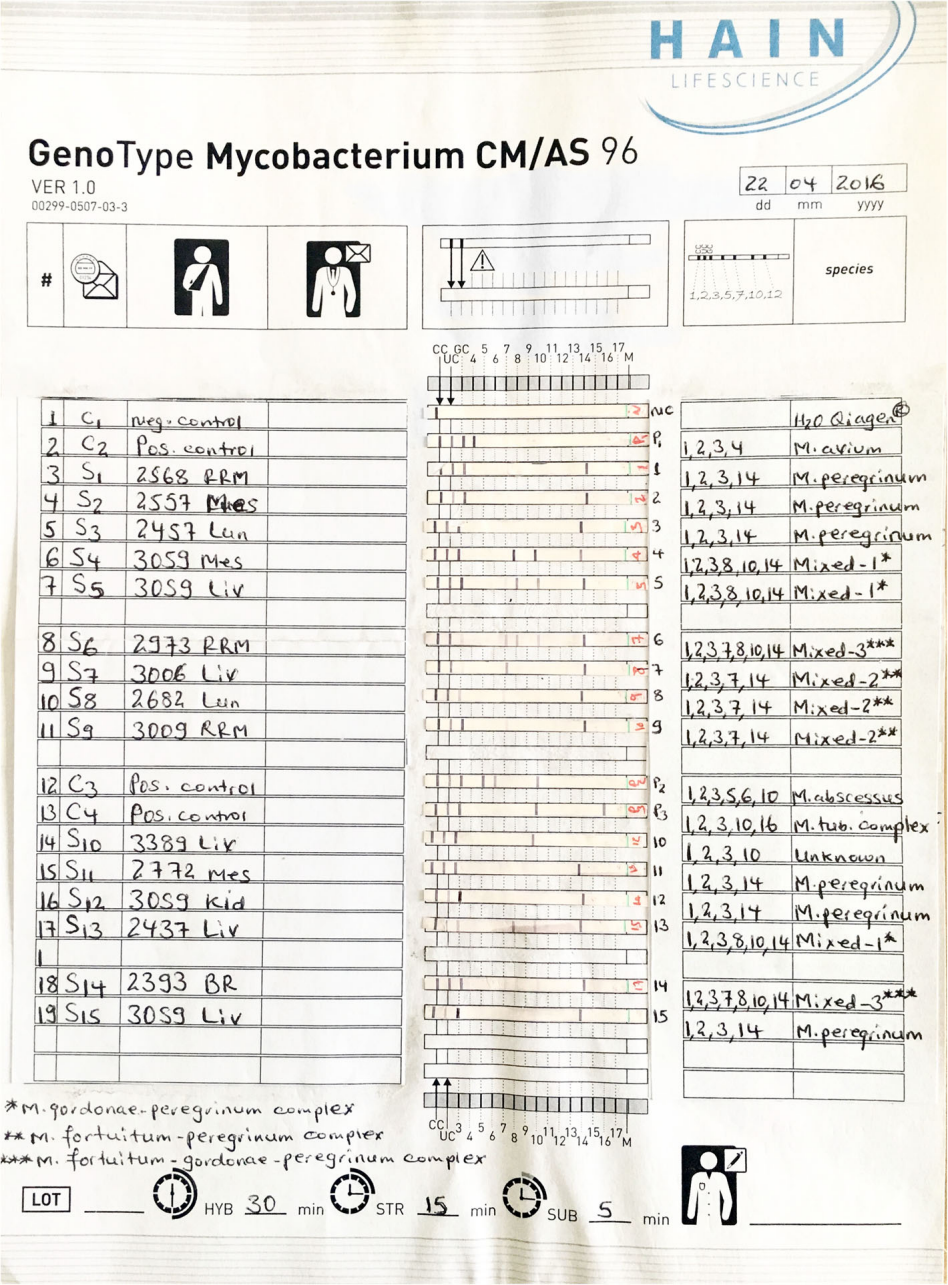
Nontuberculosis mycobacteria species identified from cattle tissue containing TB-like lesion(s). *Mixed-1: *M. gordonae-peregrinum*; **Mixed-2: *M. fortuitum-peregrinum*; ***Mixed-3: *M. fortuitum-gordonae-peregrinum*; fifteen of the 17 isolates with acid fast bacilli showed bands at 1030 bp by Gel electrophoresis and identified as nontuberculosis mycobacteria (NTM). Further characterization by GenoType® mycobacterium CM showed 14 of the 15 NTMs defined at the species level and the remaining 1 NTM (Sample code: S_10_) could not be interpreted

Further characterization of the 15 NTM by using Genotype Mycobacterium CM assay revealed that 14 isolates identified at the species level and 1 NTM could not be interpreted even if it has a band pattern of 1, 2, 3 and 10 (Fig. 2). Among the 14 isolates with defined NTM species, 6 isolates were recognized as *M. peregrinum*, and the remaining 8 represented mixed species including *M. fortuitum-peregrinum* (3 isolates), *M. gordonae-peregrinum* (3 isolates), and *M. fortuitum-gordonae-peregrinum* (2 isolates).

## Discussion

The overall prevalence of bTB from gross suspected TB lesion(s) in the present study was 2.78% which is comparable to 2.7% reported by Bekele and Belay [10], but lower than other findings ranging from 3.5% to 10.2% [7–9, 11–15, 25]. These variations could be explained by many factors including differences in the disease status in the animal populations, the sample size and the type of production system from where the slaughtered cattle were originated. Breed of animals that are slaughtered in the abattoirs and subjective differences in identifying TB lesions could also be considered for the disparities observed. The low prevalence of bTB in this study could be explained by the fact that the vast majority of cattle in the current study were Zebu and from non-intensive smallholder farms as well as most of the cattle were originated from northwest Ethiopia, where the overall prevalence of bTB was reported very low [10]. Moreover, the TB-like lesions might not always be of mycobacterial origin, rather they could also be caused by other granuloma forming organisms like *Nocardia* and *Corynebacterium* species [26], parasites and other non-specific reactions [27, 28].

The overall culture yield of AFB from visible lesions in the present study was 17.7%, which is slightly higher than 11% reported previously in Ethiopia [7], but lower proportion when it is compared to 38.1% recorded in Jimma Municipality Abattoir, southwest Ethiopia [10]. The observed differences could also be attributed to the subjective differences in identifying TB lesions, which were subjected to ZN staining microscopic examination across the study sites.

Different NTM species were identified in this study from isolates with positive AFB, notably *M. fortuitum*, *M. gordonae* and *M. peregrinum*. The NTM species such as *M. fortuitum* and *M. gordonae* are so ubiquitous that they have previously been recovered from cattle in Ethiopia [7, 29], and human, animals and the environment elsewhere in Africa [28, 30–32]. *Mycobacterium peregrinum,* which is a rapidly growing, ubiquitous and an opportunistic but potentially pathogenic NTM [33] was isolated more frequently in this study. Similar previous studies in Ethiopia [7] and Zimbabwe [34] have also reported *M. peregrinum* from cattle as well. Moreover, miscellaneous human infections, more specifically skin and lung infections were also found associated with *M. peregrinum* in Japan [35] and Brazil [36], respectively. The high rate of *M. peregrinum* isolation from lesions in the present study can suggest that this species of NTM is abundant and has high pathogenicity to cause infection in cattle in the study area as compared to other NTM species including *M. fortuitum* and *M. gordonae.* However, the role of these NTMs in TB disease causation in cattle and their zoonotic implication is not known in our cases, and these will be the objective for further investigations. Moreover, *M. fortuitum* and *M. gordonae* have been reported to elicit reactions to purified protein derivative bovine based skin tests in cattle [37]. As a result the isolation of these species in the present study emphasized further studies as mycobacteria other than *M. bovis* may interfere with current bTB diagnostic tests and ensuing in false positive test results [38].

## Conclusion

This study has isolated NTMs, notably *M. fortuitum, M. gordonae and M. peregrinum* from TB-like lesions of grazing cattle, and these findings suggest an important role of NTM in causing lesions in cattle. However, the pathogenesis of NTM species in cattle, the epidemiology (including sampling from environmental sources such as water and soil), their interactions with bTB and the zoonotic link between animal and humans is not known and needs further studies.

## Abbreviations

AFB: Acid fast bacilli
Bp: Base pair
bTB: Bovine tuberculosis
CIDT: Comparative intradermal tuberculin
CM: Common mycobacteria
LJ: Lowenstein Jensen
MTBC: Mycobacterium tuberculosis complex
NTM: nontuberculosis mycobacteria
PCR: Polymerase chain reaction
PPD-B: Purified protein derivative bovine
TB: Tuberculosis
ZN: Ziehl–Neelsen
